# The Association Between Force-Velocity Relationship in Countermovement Jump and Sprint With Approach Jump, Linear Acceleration and Change of Direction Ability in Volleyball Players

**DOI:** 10.3389/fphys.2021.763711

**Published:** 2021-11-18

**Authors:** Jernej Pleša, Žiga Kozinc, Nejc Šarabon

**Affiliations:** ^1^Faculty of Health Sciences, University of Primorska, Izola, Slovenia; ^2^Andrej Marušič Institute, University of Primorska, Koper, Slovenia; ^3^Department of Human Health, InnoRenew CoE, Izola, Slovenia; ^4^Laboratory for Motor Control and Motor Behavior, S2P, Science to Practice, Ltd., Ljubljana, Slovenia

**Keywords:** volleyball, force-velocity profiling, vertical jump, sprint, CoD

## Abstract

The force-velocity (FV) relationship allows the identification of the mechanical capabilities of musculoskeletal system to produce force, power and velocity. The aim of this study was to assess the associations of the mechanical variables derived from the FV relationship with approach jump, linear sprint and change of direction (CoD) ability in young male volleyball players. Thirty-seven participants performed countermovement jumps with incremental loads from bodyweight to 50–100 kg (depending on the individual capabilities), 25-m sprint with split times being recorded for the purpose of FV relationship calculation, two CoD tests (505 test and modified *T*-test) and approach jump. Results in this study show that approach jump performance seems to be influenced by maximal power output (*r* = 0.53) and horizontal force production (*r* = 0.51) in sprinting, as well as force capacity in jumping (*r* = 0.45). Only the FV variables obtained from sprinting alone contributed to explaining linear sprinting and CoD ability (*r* = 0.35–0.93). An interesting finding is that sprinting FV variables have similar and some even stronger correlation with approach jump performance than jumping FV variables, which needs to be considered for volleyball training optimization. Based on the results of this study it seems that parameters that refer to horizontal movement capacity are important for volleyball athletic performance. Further interventional studies are needed to check how to implement specific FV-profile-based training programs to improve specific mechanical capabilities that determine volleyball athletic performance and influence the specific physical performance of volleyball players.

## Introduction

Vertical jumping, quick accelerations, and change of direction (CoD) maneuvers are crucial components of volleyball game (Künstlinger et al., 1987; Giatsis, 2001). The higher a player is able to jump, the greater his/her potential for successful performance in offensive and defensive actions (Forthomme et al., 2005). Due to its court, volleyball requires very short-distance sprints and quick and agile movements for successful performance in defensive actions (Cengizel, 2020). The performance of a volleyball player is directly affected by the capacity of producing power in jumping and sprinting actions (Sattler et al., 2015; Schons et al., 2019). Different forms of jumps are used to evaluate the jumping power (unilateral and bilateral, vertical and horizontal) with the use of different measurement devices. CoD ability, which is underlying agility (Sheppard and Young, 2006), is usually evaluated with various tests of changes of direction in the horizontal plane (Nimphius et al., 2018), while acceleration ability is evaluated with sprint times on different distances (Kumagai et al., 2000). With more analytical approach and biomechanical testing, we can evaluate these abilities throughout more complex forms of performance tests. Those tests provide a more detailed insight into the athlete’s neuromuscular capacity and thus offer the opportunity for training optimization.

The force-velocity (FV) profiling has recently been proposed as a tool to identify the neuromuscular capabilities of athletes and to optimize the training (Jiménez-Reyes et al., 2017a). The FV relationship allows to characterize the mechanical capabilities of musculoskeletal system to produce force, power and velocity (Jaric, 2015; Samozino et al., 2016). Since the first study on the topic (Hill, 1937) it has been known that the FV relationship of individual muscles is approximately hyperbolic, while the novel studies show that FV relationship of multi-joint performance tasks is quasi-linear (Bobbert, 2012; Sánchez-Medina et al., 2014; Jaric, 2015; Sreckovic et al., 2015; Zivkovic et al., 2017). This allows using simple linear model to calculate the maximal theoretical force (i.e., the F-intercept; F_0_), maximal theoretical velocity (V-intercept; V_0_) and maximal power (P_max_ = F_0_ ⋅ V_0_/4) (Jaric, 2015). The *X* and *Y*-axis intercepts (F_0_ and V_0_) determine the slope of the FV relationship, which presents the FV mechanical profile (i.e., the individual ratio between force and velocity qualities). Steep FV relationship is reflecting that the individual is better at generating high forces at low velocities (i.e., force dominance), and vice versa if the FV relationship is less steep, the individual is better at producing high velocity at lower force (i.e., velocity dominance) (Jaric, 2015; Jiménez-Reyes et al., 2017a).

Due to its simplicity and cost-effectiveness, the FV profiling is often applied to different multi-joint movement tasks such as vertical jump (Jiménez-Reyes et al., 2017a) and sprint (Samozino et al., 2016). In addition to F_0_, V_0_, P_max_ and the slope of the FV relationship, the FV profiling in sprint allows the evaluation of the ability to produce force in horizontal direction in the acceleration phase (Samozino et al., 2016). In addition to the variables mentioned above, we can also evaluate the sprinting mechanical efficiency (maximal ratio of horizontal-to-resultant force, RF) (Morin et al., 2011). The literature suggests that FV profile can provide meaningful data to implement individualized training programs (Morin and Samozino, 2016; Jiménez-Reyes et al., 2017a). Maximal strength training increases the ability of muscles to produce force (and thus increases F_0_), while training in high velocity conditions (i.e., plyometric training) increases the V_0_ (Jiménez-Reyes et al., 2019). Vertical jump height largely depends on the maximal power output (Markovic and Jaric, 2007). Nevertheless, by changing the slope of the FV relationship, we can improve the jumping performance independently of the changes in maximal power (Samozino et al., 2012; Jiménez-Reyes et al., 2017a), which indicates the practical applications of the FV relationship.

Despite the fact that FV is well documented in the scientific literature, the question arises about its association with sport-specific movement tasks. Most of the studies have focused mainly on the explanation of the biomechanical characteristics of the FV relationship, while its direct relationship with athletic performance has not been investigated much. The optimal FV profile, well-established in jumping tasks (Samozino et al., 2012), may not transfer to optimal performance in other tasks. For instance, it has been reported that the CoD ability is related to F_0_ and P_max_ of the FV profile, while the parameters of the FV relationship in the vertical jump showed only few small correlations with CoD (Baena-Raya et al., 2020). In terms of correlations of FV profiles across tasks, only high correlations between P_max_ in sprint and P_max_ in jumping, and moderate correlations between V_0_ in sprint and V_0_ in jumping were reported (Marcote-Pequeño et al., 2019). The results of this study suggest that P_max_ could present a general measure of lower limb capacity, while F_0_ and V_0_ are more specific to the movement task. To the best of our knowledge, only one study to date have examined the association between FV relationship and athletic performance of volleyball players (Baena-Raya et al., 2021). The authors reported strong correlations between F_0_ in jumping FV relationship (*r* = 0.81–0.82) and V_0_ in sprinting FV relationship (*r* = 0.70–0.72) with ball speed in the volleyball spike and serve. The mechanical variables F_0_ in jump and V_0_ in sprint individually explained 20–36% variance of ball speed.

From the perspective of physical capabilities, the main components for successful volleyball performance are vertical jumps, quick accelerations and CoDs (Künstlinger et al., 1987; Giatsis, 2001). Those capabilities are often addressed in the training process to improve physical performance of the players. Those capabilities are usually tested through conventional performance tests (i.e., approach jump, different variants of CoD test, and linear acceleration on different distances), which offer only limited amount of information for further training adjustments. Based on that the aim of this study is to check for correlations between individual physical capabilities and FV relationship in similar movement task, to get a deeper insight into selected physical capabilities of volleyball players. This could provide a more detailed insight into selected volleyball specific physical capabilities, which is overlooked in conventional testing, and thus could potentially help with guiding training related decision-making.

The purpose of this study was to examine the relationship between FV profile obtained from linear sprint and vertical jump tasks with CoD performance (*T*-test, 505 test, CoD deficit), linear sprinting ability, and volleyball specific approach jump performance, on a sample of young male volleyball players. With these performance tests, we want to cover the key components of the volleyball movement (Forthomme et al., 2005; Hedrick, 2007). If FV relationship is associated with sports performance, it could be used to guide training-related decision making for improving performance of specific movement tasks.

## Materials and Methods

### Participants

For this study, we recruited 37 young male volleyball players (age: 21.8 ± 3.9 years; body height: 188.7 ± 7.1 cm; body mass: 82.2 ± 9.2 kg). The study took place 1 week after the formal end of the volleyball season (end of May 2021). Players were asked to not perform any resistance exercise or very exhaustive training in general, for 2 days before the measurements. They were also asked to maintain their normal nutritional habits and abstain from alcohol drinking 2 days before the measurements. All the players have been competing in first or second division of the national league. They reported to be involved in regular training for 10.9 ± 4.1 years, to attend 5.7 ± 1.2 training sessions per week and to regularly perform full body resistance exercises at least twice a week. The inclusion criteria were the absence of injuries at the time of testing and absence of any other medical diseases. All participants were informed about the experimental procedures and were required to sign an informed consent form before taking part in the experiment. For underage participants, their parents or legal guardians signed the consent on their behalf. The experiment was approved by Republic of Slovenia National Medical Ethics Committee (approval no. 0120–99/2018/5) and was conducted in accordance to the Declaration of Helsinki.

### Study Design

This was a cross-sectional study, conducted in a single visit. The participants had been performing the testing procedures as part of their regular routine assessments, therefore, no separate familiarization session was conducted. The participants performed a warm-up, consisting of 10 min of light running on an indoor track, 5 min of dynamic stretching, 5 min of bodyweight resistance exercises (squats, lunges, push-ups) and 3 min of activation exercises (vertical jumps and short-distance sprints). Then, they completed (a) assessments of FV relationship in countermovement jump, (b) 25-m linear sprint for FV relationship assessment and performance testing (5-, 10-, 15-, and 25-m sprint) and c) performance tests (modified *T*-test, 505 test and approach jump). The breaks between the tasks were at least 5 min. The order of the performance tasks was randomized. In all tasks, the average of the repetitions of each test was considered for further analyses.

### Assessment of Force-Velocity in Countermovement Jump

Vertical jumps were performed on a piezoelectric force plate (Kistler, model 9260AA6, Winterthur, Switzerland). Before jumping assessment, we measured participants leg length (form superior anterior iliac spine to big toe, with leg being fully extended), and vertical length form the floor to the superior anterior iliac spine, with knee flexed to 90°. This height was used as a reference point for the depth of the jump, which was monitored by the examiner. Participants performed vertical jumps with additional loads. External load to the subject was applied with the use of Olympic barbell. The load range was from the jump without additional load (body weight jump with plastic stick), to the maximum load with which the participants could perform safe and technically correct jump, or until the height of the jump fell below 10 cm (García-Ramos et al., 2018). Additional loads were progressed by 10 kg, with the exception of the first loaded jump, which was performed with Olympic barbell (20 kg). Each jump task was performed 2 times, with 2 min break between progressive loads. Ground reaction force data were recorded at sampling rate of 1,000 Hz. The signals were automatically processed by the manufacturer’s software (MARS, Kistler, Winterthur, Switzerland) by a moving average filter with a 5 ms window. The average value of the two repetitions was used for the analysis. We obtained the average force and average velocity data for each individual load. The FV relationship was calculated with the linear regression (force data on the *X*-axis and velocity data on the *Y*-axis). The following variables were calculated from the FV relationship: F_0_ – maximal theoretical force (N/kg), V_0_ - maximal theoretical velocity (m/s, m/s/kg), P_max_ – maximal power (W, W/kg), slope of the FV relationship (N.s/m/kg). Using the calculations introduced by the Samozino group (Samozino et al., 2012; Jiménez-Reyes et al., 2017b), we also calculated the deviation from the optimal FV profile (based on the FV relationship data, leg length and squat depth).

### Assessment of Force-Velocity in Sprint

Using 5 pairs of timing gates (Brower Timing Systems, Draper, UT, United States), we collected 0–5, 0–10, 0–15, and 0–25 m sprint times. The participants began each sprint 50 cm behind the start line, to prevent early triggering. This meant that the body was already moving forward when the timing started. Therefore, we applied a correction of 0.5 s, as recommended in the literature (Haugen et al., 2012). A standing start was used, and subjects were free to choose their front leg, which was kept constant across repetitions. Subjects were instructed to sprint from the start line through all sets of timing gates as fast as possible. Subjects performed five trials, with 2 min break between repetitions. FV relationship was calculated based on the split time data, body mass and body height, using the Excel templates designed based on the Samozino’s simplified method (Samozino et al., 2016). Sprint split times were also used as performance indicators (5, 10, 15, and 25 m), with the 0–10 m time also used for COD deficit calculation (see section “Change of Direction Performance”).

### Change of Direction Performance

The CoD assessment involved two tests (modified *T*-test and 505 test). For both tests we used single-beam laser timing gates (Brower Timing Systems, Draper, UT, United States). The gates were positioned at the hip level and recorded the times to the nearest 0.001 s. The participants began each task 30 cm behind the start line, to prevent early triggering, and began the tests at their own will. First, the participants performed the modified *T*-test, which is similar to the traditional *T*-test, but with approximately twofold shorter total distance (Sassi et al., 2009). Two warm-up repetitions with submaximal effort were performed first, followed by three test repetitions, with 2 min breaks in between.

Next, the 505 test was performed, using the same timing gates set-up. The participants were instructed to sprint to a line which was marked 15 m from the start line (with timing gates positioned 10 m form the start line) and place left or right foot on the line, turn for 180° and sprint back 5 m through the timing gates again. Three repetitions were performed for each leg in an alternating order, with 1 min breaks between the repetitions. In addition, we calculated the CoD deficit, which was recently suggested as a more isolated measure of CoD performance (Nimphius et al., 2016). In brief, the CoD deficit represent the additional time that an athlete requires to complete a CoD task compared to a linear acceleration of equal distance (Nimphius et al., 2016). Thus, to obtain the CoD deficit in 505 test, we subtracted 0–10 m sprint times (see section “Assessment of Force-Velocity in Sprint” for details) from the 505 test times.

### Vertical Jump With Approach

Vertical jumps with approach are often used in volleyball due to their resemblance to spike jump (Sorenson et al., 2010). Before the tests, the standing reach was measured with the dominant arm reaching overhead, while participants were facing the wall. Jumping reach was measured with measurement tape placed on the basketball board. Before each jump, participants chalked their fingertips for more precise detection of the jumping reach. The difference between standing reach and jumping reach presents the height of the jump. By utilizing a normal spike approach the athlete jumps for height and touches as high as possible on the measure tape at the basketball board. The subjects were instructed to perform the jumping procedure in the way that they found most convenient, similar to their personal technique during a volleyball practice. Each participant performed two warm-up trials at submaximal effort and three testing attempts, with 1 min breaks in between. Measurements were taken to the nearest cm. The difference between standing reach and jumping reach was calculated and taken for further analyses.

### Statistical Analysis

The data were analyzed with SPSS (version 25.0, SPSS Inc., Chicago, United States). Descriptive statistics are reported as mean ± standard deviation, minimum and maximum values. The normality of the data distribution was verified with Shapiro-Wilk tests (all *p* < 0.128), therefore, parametric statistics was used. Correlations among FV variables and performance variables were assessed with Pearson’s correlation coefficients and interpreted as negligible (<0.1), weak (0.1–0.4), moderate (0.4–0.7), strong (0.7–0.9), and very strong (>0.9) (Akoglu, 2018). Multiple linear stepwise regressions were done with performance variables as dependent variables and FV relationship variables as candidate predictors. The successive predictors were included in the model if they statistically significantly (*p* < 0.05) contributed to the proportion of explained variance in performance variables. The threshold for statistical significance was set at *p* < 0.05.

## Results

**FIGURE 1:**
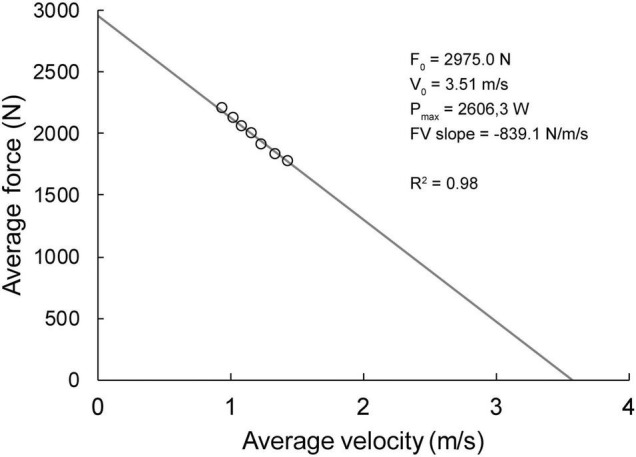
Force-velocity relationship data for representative participant.

Figure 1 depicts a representative FV profile for one participant. The descriptive statistics for all variables are presented in Table 1. The correlations among FV relationship variables and performance outcomes are available in Table 2.

**TABLE 1:** Descriptive statistics for all outcome variables.

	Outcome variables	Mean	*SD*	Minimum	Maximum
Performance indicators	Approach jump (cm)	77.07	9.40	53.67	94.67
	*T*-test (s)	5.37	0.25	4.89	6.00
	505_Left (s)	2.34	0.12	2.13	2.71
	505_Right (s)	2.31	0.12	2.09	2.57
	5m_AVG (s)	1.53	0.06	1.40	1.70
	10m_AVG (s)	2.24	0.09	2.05	2.42
	15m_AVG (s)	2.91	0.11	2.73	3.27
	25m_AVG (s)	4.14	0.15	3.83	4.62
FV in vertical jump	F_0_ (N/kg)	33.00	4.51	25.55	44.65
	V_0_ (m/s/kg)	0.05	0.02	0.03	0.10
	P_ma_ (W/kg)	33.95	7.02	21.61	53.27
	FV slope (N.s/m/kg)	−7.99	2.92	−13.51	−3.24
	FV_opt_ slope	−18.80	2.95	−28.37	−15.84
	FV imbalance (%)	43.42	18.55	2.00	82.00
FV in linear sprint	F0 (N/kg)	6.16	0.64	4.67	7.72
	V0 (m/s)	9.67	0.89	7.72	11.70
	P_max_ (W/kg)	14.79	1.40	11.26	17.75
	FV slope	−0.65	0.11	−0.92	−0.41
	RF_max_ (%)	0.41	0.02	0.36	0.45
	DRF (%)	−0.06	0.01	−0.09	−0.04
	V_opt_ (m/s)	4.84	0.45	3.86	5.85
	Max speed (m/s)	8.65	0.52	7.25	9.76

*SD, standard deviation; F_0_, maximal theoretical force; V_0_, maximal theoretical velocity; P_max_, maximal power; FV, force-velocity relationship; FV_opt_, optimal slope of the force-velocity relationship; FV Imbalance, deviation from the optimal FV relationship for power production in the vertical direction; RF_max_, ratio between vertical and horizontal ground reaction force; DRF, decrease of RF_max_.*

**TABLE 2:** Correlations between outcome variables of FV relationship and performance measures.

		Approach jump	*T*-test	505 left	505 right	CoDD left	CoDD right	5 m sprint	10 m sprint	15 m sprint	25 m sprint
FV jump	F_0_	0.45**	–0.18	–0.15	–0.06	–0.12	–0.02	–0.03	–0.05	–0.03	–0.16
	V_0_	0.09	–0.02	0.19	0.18	0.20	0.16	–0.08	–0.02	0.07	0.01
	P_max_	0.02	–0.09	0.01	0.18	0.05	0.18	–0.14	–0.08	0.04	–0.10
	FV slope	−0.18	–0.06	0.09	0.08	0.11	0.09	–0.05	–0.04	0.01	0.01
	Imbalance 90°	0.16	–0.10	–0.21	–0.21	–0.22	–0.18	0.06	0.02	–0.07	–0.07
FV sprint	F_0_	0.35*	−0.42**	–0.21	0.04	−0.35*	0.54**	−0.89**	−0.78**	−0.49**	−0.48**
	V_0_	0.13	–0.08	–0.16	−024	–0.15	–0.21	0.26	–0.01	−0.33*	−0.43**
	P_max_	0.53**	−0.58**	−0.38*	–0.19	0.23	0.38*	−075**	−0.86**	−0.89*	−0.93**
	FV slope	−112	0.21	0.02	–0.18	−0.30*	−0.45**	0.66**	0.49**	0.06	0.05
	RF_max_	0.51**	−0.52**	−0.32*	–0.10	0.32*	0.48*	−0.93**	−0.91**	−0.80**	−0.76**
	DRF	−0.08	0.19	0.012	–0.18	–0.29	−0.44*	0.63**	0.43**	0.02	0.01
	V_opt_	0.13	–0.08	–0.16	–0.24	–0.15	0.21	–0.01	–0.01	–0.33	−0.43**
	Maximal speed	0.27	–0.19	–0.25	–0.28	–0.09	–0.11	–0.21	–0.21	−0.56**	−0.61**

*p < 0.05; **p < 0.01; CoDD, change of direction deficit; F0, maximal theoretical force; V_0_, maximal theoretical velocity; P_max_, maximal power; FV, force-velocity relationship; FV_opt_, optimal slope of the force-velocity relationship; FV Imbalance, deviation from the optimal FV relationship for power production in the vertical direction; RF_max_, ratio between vertical and horizontal ground reaction force; DRF, decrease of RF_max_.

The approach jump height was in moderate correlation with F_0_ in countermovement jump (*r* = 0.45, *p* < 0.01) and in moderate to high correlations with F_0_ in sprint (*r* = 0.35, *p* = 0.022), P_max_ in sprint (*r* = 0.53; *p* < 0,001), and RF_max_ (*r* = 0.51; *p* = 0.001). In linear regression model, 62.2% of the variance in approach jump was explained by sprint P_max_, Jump F_0_, and Jump V_0_ (21.3% and 36.0% with sprint P_max_, and sprint P_max_ + Jump F_0_, respectively).

Modified *T*-test was in moderate to high negative correlation (*p* < 0.01) with F_0_, P_max_ and RF_max_ in sprint (*r* = −0.42 to −0.58). The negative correlation means that the subjects with faster *T*-test times exhibited higher values of FV outcome variables. In linear regression model, 34.7% of the variance in modified *T*-test times was explained with sprint P_max_, with no additional contribution of other variables.

The performance of 505 test on the left leg was in moderate negative correlation (*p* = 0.011–0.032) with RF_max_ (*r* = −0.32) and sprint P_max_ (*r* = −0.38). The direction of these correlations implied that 505 test performance on the left leg improved with higher values of reported FV outcome variables. In linear regression model, 13.7% of the variance in 505 time on the left leg was explained with sprint P_max_, with no additional contribution of other variables. 505 test performance on the right leg was not correlated with any of the FV variables (*p* = 0.059–0.724). CoD deficit on the left leg (*p* = 0.045–0.021) was in moderate correlation with sprint F_0_ (*r* = 0.35), slope of the FV relationship in sprint (*r* = −0.30) and RF_max_ (*r* = −0.32). CoD deficit on the right leg was in moderate to high correlation with F_0_ (*r* = 0.54), P_max_ (*r* = 0.38), slope of the FV relationship (*r* = −0.45), RF_max_ (*r* = 0.48), and DRF (*r* = −0.44). Sprint F_0_ explained 11.6% and 34.1% of the variance CoD deficit on left and right legs, respectively. There were no significant correlations between the outcome variables of FV variables in vertical jump and CoD performance or CoD deficit.

The 5 and 10 m sprint time was in moderate to very strong correlation (*r* = 0.43–0.93; *p* < 0.05) with F_0_ (negative correlation), P_max_ (negative correlation), slope of the FV relationship, RF_max_ (negative correlation), and DRF. In linear regression model, RF_max_ alone explained 85.6 and 78.2% of the variance in 5 and 10 m sprint times, respectively, with additional contribution of F0 for 5 m time (88.2% of the variance explained). The 15 and 25 m sprint times were in moderate to very strong negative correlation (*r* = −0.33 to −0.93; *p* < 0.05) with RF_max_, V_opt_ and maximal speed. P_max_ alone explained 71.9% of the variance in 15 m sprint and 87.7% of the variance in the 25 m sprint, with no contribution from other variables.

**FIGURE 2:**
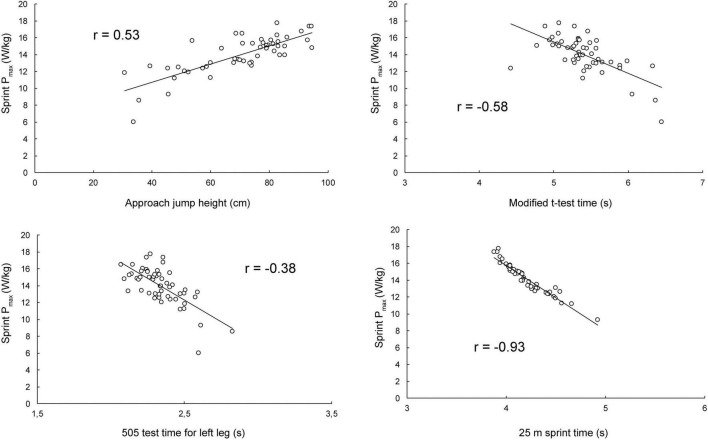
Relationships between sprint maximal power and approach jump, modified *t*-test, 505 test and 25-m sprint performance.

Overall, the sprint Pmax showed the highest associations with performance measures. Four selected relationships with sprint Pmax are depicted on Figure 2.

## Discussion

The purpose of this study was to examine the association between the FV relationship variables obtained in linear sprint and vertical jumps, and volleyball-specific approach jump performance, linear sprint and CoD ability (modified *T*-test, 505 test and CoD deficit) on a sample of young male volleyball players. Our results show that the only correlation between FV relationship in vertical jump and performance variables was between F_0_ and approach jump height. While in sprinting FV relationship we found: (a) weak to moderate correlations with approach jump, (b) weak to moderate negative correlations with CoD performance, except of correlations with right leg in 505 test, and (c) negative weak to very strong correlations with sprinting ability on different distances. In regression models, sprinting P_max_ was included as a predictor of approach jump performance and *T*-test performance, while RF_max_ was a predictor of sprinting ability on all distances. Therefore, approach jump performance seems to be influenced both by sprint and jumping FV profiles, while FV sprint variables alone contributed to explaining linear sprinting and CoD ability.

To our knowledge, only one study to date have examined the association between FV relationship and specific sport performance of volleyball players (Baena-Raya et al., 2021). The main finding of their study was that the *F*_0_ obtained from vertical jumping and the *V*_0_ obtained from sprinting, were strongly associated with both spike and serve ball speeds. These mechanical variables were able to explain approximately 20–36% of the variability in spike and serve speeds. Along with results in our study, those observations could help coaches implement specific FV profile-based training programs to improve specific mechanical capabilities that determine specific athletic performance, such as approach jump height, linear acceleration, and CoD ability, as well as to improve specific volleyball performance such as spike and serve ball speed in male volleyball players. The specific finding of our study is that performance proxies (approach jump, CoD tests, and sprinting) were related mostly to sprint-based FVP variables. It seems that producing high horizontal power is one of the paramount abilities underpinning sports performance. It has already been shown that maximal horizontal power is key determinant of linear sprinting performance (Morin et al., 2012). This study highlights that sprint P_max_ is also related to superior CoD performance and jumping ability. On the other hand, approach jump performance appears to be related to vertical force and horizontal power production capacity.

A somewhat surprising finding in this study is that sprinting FV variables have similar and some even stronger correlation with approach jump performance than jumping FV variables, which at first glance does not agree with the principle of specificity and force vector theory. Briefly, the direction of the resistance force vector relative to the body play a role in transference to sport specific performance (axially resisted movements appear to better transfer to vertical-based movements such as vertical jump and anteroposterior resisted movements appear to better transfer to horizontal-based activities such as linear sprint) (Randell et al., 2010; Contreras et al., 2017). Thus, it is important to consider the direction of movement, both in terms of training optimization and as well as in terms of relevance of biomechanical testing for analyzing sport-specific movement performance. Approach jump consists of short running approach, following by two-legged jump. One leg is usually in front of the other, with foot directed slightly inward, to emphasize vertical direction of the jump and prevent too much horizontal flight (Prsala, 1982; Honish, 2005). This movement is quite complex, requiring good movement coordination (Ciapponi et al., 1996; Honish, 2005). The purpose of approach is to create high horizontal force, which is later transferred into vertical direction to jump as high as possible. The penultimate step (the last step of the approach) is the longest, and is performed in explosive manner. Studies reported the importance of penultimate step for approach jump performance (Liu et al., 2001). Longer and more explosive penultimate step has larger horizontal velocity and momentum, which is reflected in higher approach jump (Liu et al., 2001). In this study, we did not control the penultimate step length, so we cannot explain the obtained correlations to be actually dependent on penultimate step. These findings could present an important consideration for volleyball training optimization. Volleyball players on average perform 250–300 explosive actions in a single game, with more than half of these actions being vertical jumps (Mattes et al., 2018). Furthermore, a study looking into the association between volleyball training duration and number of jumps reported, that players on average performed 63 jumps per training hour (Bahr and Bahr, 2014). Thus, overuse injuries are common in volleyball players (Reeser and Bahr, 2008; Kilic et al., 2017). In regard to FV relationship, a recent study has reported that sprint F_0_ might be associated with increased hamstring injury risk (Edouard et al., 2021). Further studies are needed to determine if FV profiling could be also used to determine the risk of injuries in volleyball.

Sprinting FV profile provides information on how effectively the athlete applies high levels of force onto the ground at different contraction velocities (Hicks et al., 2020). This is especially interesting for volleyball players, who need to accelerate their own body quickly over a very short distance, in order to perform a fast tempo attack, spike after a previous action, dig or transition from defensive action (Fuchs et al., 2019). Results in this study, show that correlation between F_0_ and RF_max_ in sprinting FV with sprint performance is dropping with increasing distance. This indicates an importance of maximal force production and ability of horizontal force application for acceleration performance over short distance. Conversely, the correlations between sprint performance and P_max_ increases with sprint distance. The FV relationship explained little variance in CoD performance ability. A reason for that could be in quite homogeneous CoD performance results. In addition, CoD ability is a complex movement task that requires good movement coordination (Sheppard and Young, 2006) and is highly dependent on eccentric power (Spiteri et al., 2014; Jones et al., 2017), which is not largely reflected in FV relationship outcomes. Nevertheless, our results regarding the association between FV profiles and CoD performance are in agreement with a previous study (Baena-Raya et al., 2021), although they showed larger associations. Overall, the sprinting FV profile could be a useful assessment tool when trying maximize acceleration capabilities through training interventions, which, in turn, may translate into improved CoD performance. However, further longitudinal and experimental research is needed to confirm this hypothesis.

An interesting finding of this study is that all the participants were velocity dominant (imbalance from the optimal FV relationship in vertical jump was 2–82%; on average 43%), which means that velocity dominance in vertical jump could be: (a) a general adaptation to volleyball training, or (b) necessary predisposition to play volleyball at this level. This is in accordance with a study conducted on male and female elite volleyball players, who also showed exclusively velocity-dominant profiles (FV imbalance = 33–37%) (Broussal et al., 2016). A few other studies also reported high homogeneity toward velocity dominance of various athletes such as young ballet dancers (FV imbalance = 42–51%) (Escobar Álvarez et al., 2020), trained male track and field athletes (sprinters and jumpers; FV imbalance = 44 ± 16% for sprinters and 46 ± 14% for jumpers) (Jiménez-Reyes et al., 2014) female soccer players (FV imbalance = 65 ± 16%) (Marcote-Pequeño et al., 2019). Thus, the amount of ballistic actions such as CoDs, sprinting, jumping could explain predominance of velocity qualities. On the other hand, a study on rugby players reported mixed results considering FV dominance (Jiménez-Reyes et al., 2017a), with seven out of total forty-eight participants showing a FV profile toward force capabilities (FV imbalance = 130 ± 12%). Rugby players are usually included in heavy resistance training programs, which could explain their orientation toward force dominance in jumping FV profile. This shows that FV profile orientation is different across sports, while a very high variability between individuals is also present within each sports (Haugen et al., 2019). The correlations between FV relationship variables and performance outcome variables obtained in our study could be different if the sample would be more heterogeneous from the perspective of FV relationship dominance (i.e., including both force-dominant and velocity-dominant athletes). Consequently, it is recommended to perform correlation analysis between FV relationship in different movement tasks for each sport separately, using specific-sport performance indicators in order to better understand the potential for training optimization through monitoring and changing FV capabilities.

## Limitations

Some limitations of the study with implications for future research must be underlined. The study was conducted on a sample of well-trained male volleyball players, which means that the results should not be generalized to females and athletes from other sports, and even volleyball players of lower training status. The cross-sectional design precludes establishing any causal relationships, thus, further prospective and experimental research is needed to corroborate our results. Moreover, the variables in the study covered only a limited aspect of performance. Future studies should analyze the relationship (and changes in time) of different variables of the FV profile in other frequent actions in volleyball game. Moreover, it would be interesting to see an interventional study looking into the changes in FV relationship variables through the entire season. Fitness testing often occurs at multiple time points throughout a year for team sport athletes (pre, mid, and post-season is common) and it should not be assumed that FV relationship variables would be the same during the whole season. Finally, although a fair amount of breaks was provided between tasks and repetitions, the overall experimental protocols was relatively demanding, thus, some effects of fatigue cannot be excluded.

## Conclusion

In this study, we found that approach jump performance seems to be influenced by both, sprinting and jumping FV profiles, while FV sprint variables alone contributed to explaining linear sprinting and CoD ability. An interesting finding is that sprinting FV variables have similar and some even stronger correlation with approach jump performance than jumping FV variables, which could present an important consideration for volleyball training optimization. Evaluating FV profile in volleyball players seems to have a potential for training optimization through implementing specific FV profile-based training programs to improve specific mechanical capabilities that determine volleyball athletic performance (approach jump height, linear acceleration, and CoD ability), as well, to improve performance in specific volleyball movement tasks (spike and serve ball speed). Future studies should assess the effects of interventions with implementing specific exercises to optimize certain parameters of the FV relationship, and check if the results of the performance tests changed. Moreover, it would be interesting to see the differences in FV profile characteristics between different volleyball player roles (middle blocker, outside hitter, opposite, setter, and libero). Finally, longitudinal studies would be also desirable to see if FV profiling could be used to determine risk of the injuries in volleyball.

## Data Availability Statement

The raw data supporting the conclusions of this article will be made available by the authors, without undue reservation.

## Ethics Statement

The studies involving human participants were reviewed and approved by Republic of Slovenia National Medical Ethics Committee. The patients/participants provided their written informed consent to participate in this study.

## Author Contributions

ŽK and NŠ conceptualized the idea, overviewed the measurement procedures and administration, and finalized the manuscript. JP carried out the measurements and wrote the manuscript. JP and ŽK analyzed the collected data. All authors contributed to the article and approved the submitted version.

## Conflict of Interest

NŠ was employed by company S2P, Science to Practice, Ltd. The remaining authors declare that the research was conducted in the absence of any commercial or financial relationships that could be construed as a potential conflict of interest.

## Publisher’s Note

All claims expressed in this article are solely those of the authors and do not necessarily represent those of their affiliated organizations, or those of the publisher, the editors and the reviewers. Any product that may be evaluated in this article, or claim that may be made by its manufacturer, is not guaranteed or endorsed by the publisher.
